# Aggregate Roundness Classification Using a Wire Mesh Method

**DOI:** 10.3390/ma13173682

**Published:** 2020-08-20

**Authors:** Sung-Sik Park, Jung-Shin Lee, Dong-Eun Lee

**Affiliations:** 1 Department of Civil Engineering, Kyungpook National University, 80 Daehakro, Bukgu, Daegu 41566, Korea; sungpark@knu.ac.kr (S.-S.P.); jhjs14@knu.ac.kr (J.-S.L.); 2 School of Architecture, Civil, Environment and Energy, Kyungpook National University, 80 Daehakro, Bukgu, Daegu 41566, Korea

**Keywords:** aggregate, classification, wire mesh, roundness, tilting angle, opening size

## Abstract

Herein, we suggest a wire mesh method to classify the particle shape of large amounts of aggregate. This method is controlled by the tilting angle and opening size of the wire mesh. The more rounded the aggregate particles, the more they roll on the tilted wire mesh. Three different sizes of aggregate: 11–15, 17–32, and 33–51 mm were used for assessing their roundness after classification using the sphericity index into rounded, sub-rounded/sub-angular, and angular. The aggregate particles with different sphericities were colored differently and then used for classification via the wire mesh method. The opening sizes of the wire mesh were 6, 11, and 17 mm and its frame was 0.5 m wide and 1.8 m long. The ratio of aggregate size to mesh-opening size was between 0.6 and 8.5. The wire mesh was inclined at various angles of 10°, 15°, 20°, 25°, and 30° to evaluate the rolling degree of the aggregates. The aggregates were rolled and remained on the wire mesh between 0.0–0.6, 0.6–1.2, and 1.2–1.8 m depending on their sphericity. A tilting angle of 25° was the most suitable angle for classifying aggregate size ranging from 11–15 mm, while the most suitable angle for aggregate sizes of 17–32 and 33–51 mm was 20°. The best ratio for the average aggregate size to mesh-opening size for aggregate roundness classification was 2.

## 1. Introduction

Aggregates are necessary for manufacturing various construction materials such as concrete, mortar, brick, ballast, filter, backfill, and so forth. Natural aggregates with different shapes and sizes are obtained from rivers and seas, but they are becoming depleted. Currently, artificial aggregates are produced by crushing rock fragments from mountains. Both natural and artificial aggregates are widely used in many civil engineering structures, such as buildings, bridges, tunnels, roads, dams, among others. Moreover, a huge amount of aggregate is recycled from concrete structures and asphalt pavements, of which 85% is utilized in earthworks and road sub-bases in Korea [1]. The shape and size of aggregates contribute to their engineering characteristics such as interlocking, friction, and settlement. Rounded aggregates used in concrete give excellent performance and high strength whereas angular aggregates are utilized in road sub-bases and railway ballast for minimizing settlement and producing high interlocking [2]. Lee carried out a series of direct shear tests on recycled aggregates and showed a 4° higher friction angle for angular-shaped aggregates than rounded ones [3]. Therefore, the particle-shape classification for large amounts of aggregate is important because the aggregate’s engineering characteristics depend on its shape. Even though the shape is more important than the size for engineering purposes, the aggregates are usually classified by size through sieve analysis in practice.

The measurement of particle shape is difficult due to varying edges and irregular surfaces. Various indexes such as the particle perimeter, convexity, sphericity, and shape factor have been suggested to classify the particle shape for mainly academic purposes [4,5,6,7,8,9,10,11,12]. A comparison of these indexes and their key parameters are introduced in Table 1. The determination of such indexes seems to be difficult for practical purposes, especially for the angularity factor. Krumbein [10] and Rittenhouse [12] both presented charts of aggregate shape for classifying them based on sphericity. The abrasion of particles can be quantitatively determined using the Krumbein chart and is in the range of 0.1–0.9. The roughness of angular particle edges is 0.1 before abrasion and 0.9 afterward. The Rittenhouse chart shows the degree to which the shape of a particle approaches a sphere. The sphericity of a particle is the ratio of the surface area of a sphere of the same volume as the particle to the actual surface area of the particle. The sphericity of a very irregularly shaped particle is 0.45 whereas one that is nearly spherical is 0.97 and one that is completely spherical is 1. However, the analysis of particle shapes using the aforementioned methods is limited as they depend on the judgment of the one doing the particle classification [13]. Digital image processing can be used to quantitatively analyze the characteristics of the shape and/or the shape coefficient of an aggregate particle [14,15,16]. Furthermore, stereoscopic image analysis can be performed using two cameras to determine the surface area characteristics of aggregates [17,18,19].

The results of previous studies indicate that particle classification is usually performed on each particle rather than on the number of particles, and a practical classification method is still required in the field. Therefore, it is necessary to improve the way of evaluating the particle shape. In this study, a wire mesh method was investigated for classifying three different sizes of aggregates by controlling the tilt angle and using a suitable opening size of wire mesh. A classification chart for the wire mesh method is also proposed to simplify the classification of a large amount of aggregate.

## 2. Wire Mesh Method

### 2.1. Materials

**Figure 1:**
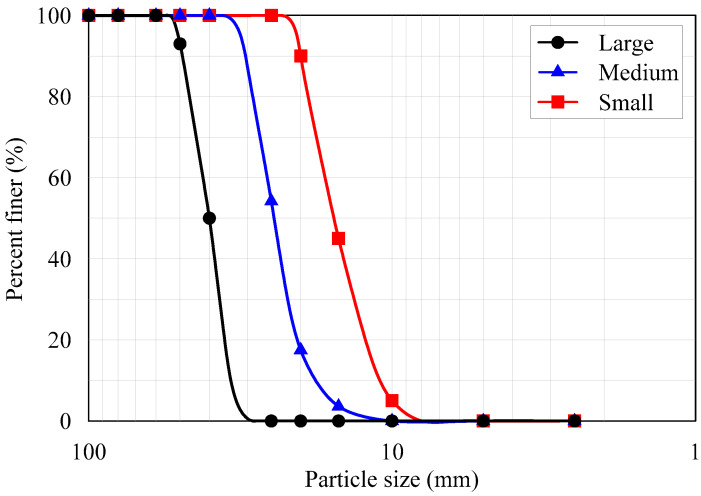
Particle size distribution curves of the three different sizes of aggregate.

Crushed aggregates used in more than 90% of Korean construction sites were used in this study. The aggregates were classified into three different sizes after performing a sieve analysis according to the KS F 2502 [20] standard. Particle size distribution curves are shown in Figure 1. Aggregate particles between 11 mm and 15 mm are termed small aggregates, those between 17 mm and 32 mm are called medium aggregates, and those between 33 mm and 51 mm are termed large aggregates. The small, medium, and large aggregates are commonly used in concrete making, road sub-base, and railroad ballast at the site, respectively [21,22].

**Figure 2:**
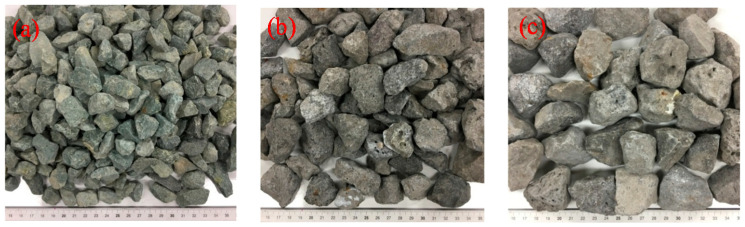
Photographs of the three different sizes of aggregate: (**a**) Small, (**b**) Medium, (**c**) Large.

**Figure 3:**
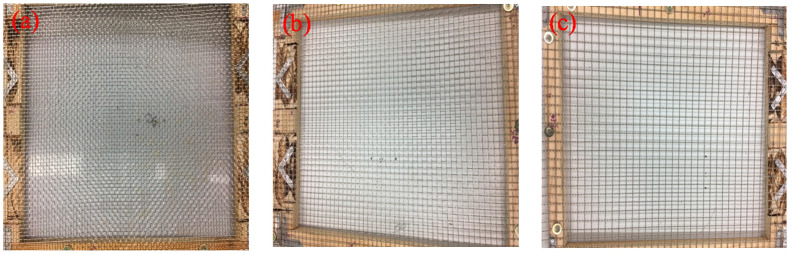
Photographs of the three different sizes of wire mesh: (**a**) 6 mm, (**b**) 11 mm, and (**c**) 17 mm.

**Figure 4:**
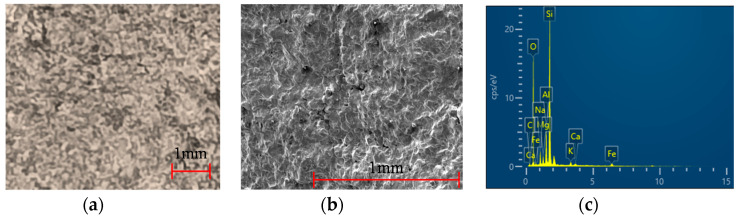
Petrographic analysis: (**a**) × 10 microscope, (**b**) × 50 microscope, (**c**) mineral components.

The three types of the aggregates (small, medium, large) are illustrated in Figure 2. In addition, three wire mesh opening sizes were selected depending on the type of aggregate, as shown in Figure 3. The aggregate particles had a variety of shapes and their color was mostly blue. The specific gravity of the aggregates is 2.7 and detailed engineering properties of the aggregates are summarized in Table 2. A wear rate indicates the reduction in the size of aggregate due to friction. The bulk densities of the each size were obtained by the test according to ASTM C 29 [23]. The results of petrographic analysis from the scanning electron microscope (SEM) were shown in Figure 4, micro structure images (a, b) and mineral component spectrum from the energy dispersive X-ray spectroscopy (EDX) (c). Petrographic analysis indicated that the aggregate is a granite-based rock containing mainly silica and alumina.

### 2.2. Wire Mesh Method

**Figure 5:**
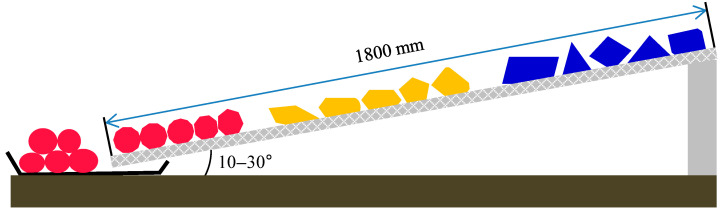
Illustration of particle-shape classification using the wire mesh method.

Herein, we suggest the particle shape classification of a large amount of aggregate using a wire mesh method. The concept of the particle shape classification using the wire mesh method is illustrated in Figure 5. The basic concept is that the greater the degree of particle roundness, the longer the moving distance while rolling on the inclined wire mesh plane. The aggregates were rolled from various tilting angles of the wire mesh that was determined by trial and error for proper classification. The aggregate particles with different sphericities were colored differently and then used for classification via the wire mesh method.

The frame containing the wire mesh was 1800 mm long and 500 mm wide. Three wire mesh plywood frames were connected with enough rolling distance depending on the sloping angle of the frame, which was a parameter considered during the experiment. When this technique is applied in the field, the size of the wire mesh frame can be modified to accommodate a suitable amount of aggregate. Suitable amounts of aggregate (1–5 kg) were poured onto the start position of the wire mesh, which was then tilted to the desired angle. The aggregates started to roll due to gravity depending on the particle shape. The wire mesh frame was inclined at various angles (10°, 15°, 20°, 25°, and 30°) to evaluate the rolling degree. The tilting angle was selected depending on the aggregate size and the wire mesh opening size. For the given aggregate and wire mesh used in this study, the majority of aggregates rolled down when the tilting angle was higher than 30°, and thus particle classification was not properly achieved. At a low tilting angle, it was difficult to classify the aggregate because only some of it moved. The aggregate that rolled and traveled the longest distance (from 1200–1800 mm) was classified as rounded, that which rolled and traveled to the middle of the wire mesh (from 600–1200 mm) was classified as sub-rounded/sub-angular, and that which remained on the inclined wire mesh without rolling or falling (from 0–600 mm) was classified as angular. These ranges were determined by trial and error.

## 3. Results and Discussion

### 3.1. Particle-Shape Classification Using a Sphericity Index

**Figure 6:**
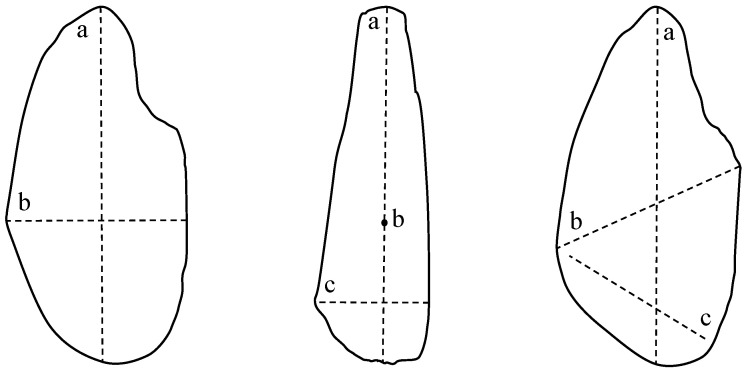
Particle dimension definition (**a**, **b**, and **c** are the longest, intermediate, and the shortest diameter).

In this study, before applying a wire mesh method the sphericity proposed by Krumbein [10] was used for classifying aggregates. The sphericity Ψ is calculated as:

(1) $$\Psi^{3}=\;\frac{\mathrm{bc}}{\;a^{2}}$$

where a, b, and c are the longest, intermediate, and shortest diameters of the aggregate particle, as shown in Figure 6. Sphericity is in the range of 0.1–0.9, and when it is close to 1, the particle shape is close to spherical, while ≤0.1 means that the particle shape is angular.

The sphericities of three different sizes of aggregate particles (11–15, 17–32, and 33–51 mm) was measured, and based on these values, the aggregate particles were classified as rounded, sub-rounded/sub-angular, or angular. The corresponding sphericity indexes were 0.8–0.9, 0.5–0.7, and 0.3–0.4, respectively. As an example, the sphericity indexes and a comparison of 30 small-sized aggregates with different sphericities were included in Table 3.

### 3.2. Particle-Shape Classification Using the Wire Mesh Method

**Figure 7:**
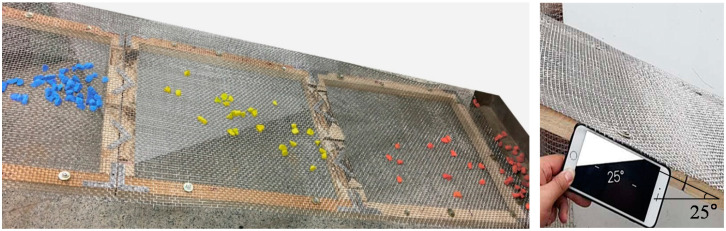
Aggregate shape classification via the wire mesh method (11–15 mm).

Three different aggregate-particle sizes of 11–15, 17–32, and 33–51 mm were used to classify the roundness of the aggregate. These aggregates had already been classified by the sphericity index to angular, sub-angular/sub-rounded, and rounded as described in Section 3.1. The wire mesh was inclined at various angles of 10°, 15°, 20°, 25°, and 30° to evaluate the rolling degree of the aggregates. When the tilting angle and opening size of the wire mesh had been optimally adjusted, the aggregates rolled and stopped on the tilted wire mesh depending on their roundness. The results showed that the angular aggregates remained on the wire mesh in the interval from 0.0–0.6 m from the top, the sub-angular/sub-rounded aggregates remained in the interval from 0.6–1.2 m, and the rounded aggregates remained in the interval between 1.2–1.8 m. For the small aggregate-particle size, some of the aggregate rolled down with a tilting angle of 20° but all the aggregates rolled down at 30°. The most suitable tilting angle for the small aggregate particles was 25°, as shown in Figure 7. For the middle- and large-sized aggregate particles, some of the aggregate rolled down with a tilting angle of 15° but all of it rolled down at 25°. The most suitable tilting angle for these aggregates was 20°. The ratio of the average aggregate size to mesh-opening size was approximately 2 for reasonable classification. When the ratio was less than 2, all of the aggregate rolled down. On the other hand, when the ratio was greater than 2, most of the aggregate particles rolled less far.

### 3.3. Comparison of Particle-Shape Classification Methods

**Figure 8:**
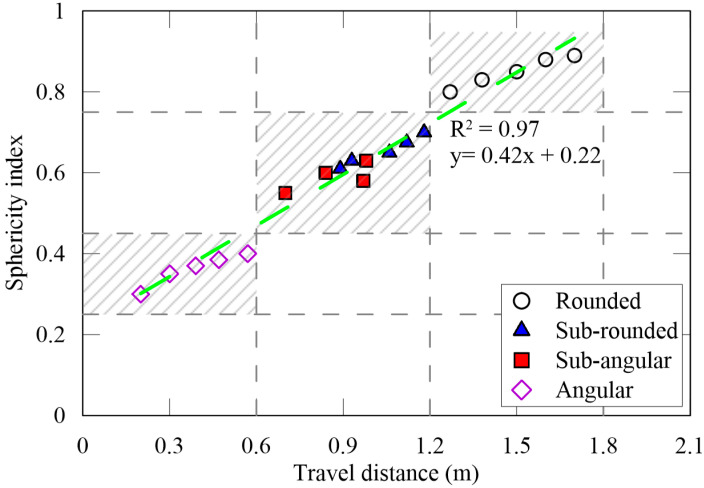
Relationship between the sphericity measurements and the wire mesh technique.

The potential usage of the wire mesh method for classifying a large amount of aggregate was evaluated via comparison with the sphericity index. The relationship between the sphericity index (y) and the travel distance (x) from the top of small-sized aggregate particles is shown in Figure 8. The correlation showed a linear relationship (y = 0.42x + 0.22) and the correlation coefficient (R^2^) is 0.97. The comparison between the sphericity and the wire mesh method showed a well-matched correlation. Similar relationships were observed for all three aggregate-particle sizes.

**Figure 9:**
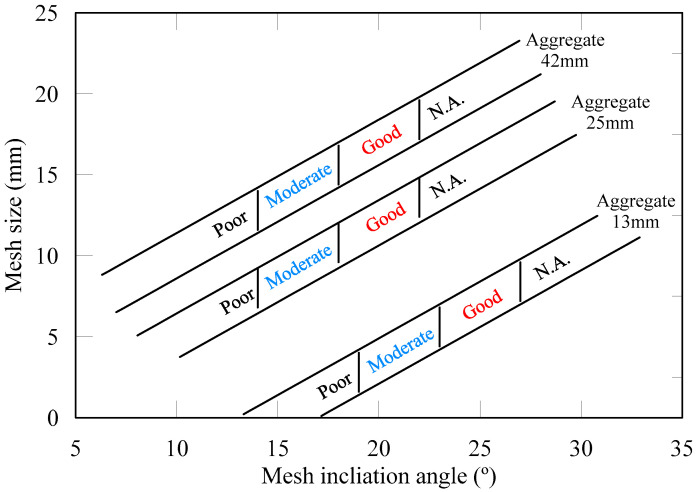
Aggregate shape classification with the wire mesh method.

The tilting angle and opening size of wire mesh needs to be determined for accurately classifying aggregates using a wire mesh method. A classification chart for the opening size and tilting angle of the wire mesh is shown in Figure 9. It can be used to select a suitable tilting angle and size of mesh. Three classification levels are suggested: good, moderate, poor, and N.A. When the average lengths of the aggregate diameter were 34 and 24 mm, the most efficient tilting angle of the wire mesh was 20° whereas, for an average length of 14 mm, the tilting angle was 25°. The greater the aggregate-particle size, the greater its self-weight, so it is expected that even with a low tilting angle, large aggregate particles can roll down due to their self-weight. For the wire mesh method, the tilting angle of the wire mesh seems to decrease as the aggregate size increases. When the ratio of the average aggregate size to the opening size of the wire mesh was 2, all of the aggregate types were properly distinguished from one another. Hence, our method can be used for aggregate-particle shape verification and classification.

## 4. Conclusions

In this study, the particle-shape classification of a large amount of aggregate was conducted using a wire mesh method. Three different types of aggregate that are usually used on construction sites were selected by size (11–15 mm, 17–32 mm, and 33–51 mm) to test the performance of the wire mesh classification. Based on the experimental results, the following conclusions can be drawn:

1. The sphericity of the three different sizes of aggregate was measured for classifying them as angular, sub-angular/sub-rounded, and rounded based on sphericity ranges of 0.3–0.4, 0.5–0.7, and 0.8–0.9, respectively.
2. The aggregates classified via sphericity were used to evaluate the wire mesh method. The opening size and tilting angle of the wire mesh were adjusted to properly classify the aggregate-particle shapes. When these factors are optimal, the angular aggregate remained on the wire frame in the interval from 0.0–0.6 m from the top, the sub-angular/sub-rounded aggregate remained in the interval from 0.6–1.2 m, and the rounded aggregate remained in the interval from 1.2–1.8 m.
3. As the tilting angle of the wire mesh increased, most of the aggregate rolled down and the classification became difficult. A suitable inclination angle of the wire mesh for the aggregate with a size range of 11–15 mm was 25°, while that for the aggregate with size ranges of 17–32 and 33–51 mm was 20°. When the opening size of the wire mesh was too small, all of the aggregate tended to roll down. On the other hand, when it was too large, none of aggregate rolled. The experimental results show that the ratio of the mesh-opening size to the average aggregate size is 1:2.
4. Classification via the wire mesh method showed similar results when using the sphericity index. Therefore, the wire mesh method can be used to classify a large amount of aggregate with different sizes and shapes at once by controlling the opening size and tilting angle of the wire mesh in practice.

## Figures and Tables

**Table 1:** Particle angularity index.

Index (Reference)	Equation	Key Parameter	Symbol
Roundness (Wadell [9])	$\frac{N}{\sum_{i=1}^{N}\frac{D}{d_{i}}}$	Corner diameter	N: number of D: diameter of largest inscribed circle d: diameter of corners
Sphericity (Krumbein [10])	$\Psi^{3}=\frac{b\times c}{\;\;a^{2\;\;}}$	Diameter (a, b, c)	Ψ: Sphericity a: The longest diameter b: The longest diameter perpendicular to a c: Second longest diameter perpendicular to a
Angularity factor, AF (Sukumaran [14])	$AF=\frac{\sum_{i=1}^{N}{\left(\beta_{i\;particle}-180\right)}^{2}\left(\frac{360^{2}}{N}\right)}{3\times 180^{2}-360^{2}/N}$	Number of sampling points and internal angle	N: Number of sampling points $\beta_{i\;particle}$: Internal angle
Volume ratio (Fei Xu [11])	$V_{B}=\frac{1}{6}\pi L_{max}^{3}$	External volume	$V_{B}$: Aggregatecircumscribed volume L: The longest length

**Table 2:** Physical properties of the used aggregates.

Bulk Density (kN/m^3^)	Water Absorption (%)	Modified CBR * (%)	Wear Rate (%)	OMC ** (%)	Porosity (%)	Contamination Content (%)	Sand Equivalent (%)
15.20 (Small) 15.32 (Medium) 15.69 (Large)	6.16	58	36.5	11.3	2–3	0.11	45

* CBR: California bearing ratio, ** OMC: optimal moisture content.

**Table 3:** Aggregate roundness classification.

Parameter	Sample (11–15 mm)									
	1	2	3	4	5	6	7	8	9	10
a (mm)	21.03	18.35	22.86	20.53	17.18	17.55	18.0	16.36	18.29	15.41
b (mm)	17.02	16.27	17.68	14.87	13.42	13.89	15.21	14.52	16.99	14.27
c (mm)	14.02	13.25	14.14	13.46	13.38	11.99	13.8	14.3	13.63	12.69
Ψ	0.8	0.9	0.8	0.8	0.9	0.8	0.9	0.9	0.9	0.9
**Parameter**	**Sample (11–15 mm)**									
	**11**	**12**	**13**	**14**	**15**	**16**	**17**	**18**	**19**	**20**
a (mm)	21.79	19.51	20.42	23.13	27.0	19.77	18.66	17.87	19.63	22.14
b (mm)	11.03	9.59	13.85	13.11	14.43	9.15	14.08	7.56	13.27	13.07
c (mm)	6.93	6.01	11.46	10.03	12.66	6.11	10.08	6.21	11.04	8.73
Ψ	0.5	0.5	0.7	0.6	0.6	0.5	0.7	0.5	0.7	0.6
**Parameter**	**Sample (11–15 mm)**									
	**21**	**22**	**23**	**24**	**25**	**26**	**27**	**28**	**29**	**30**
a (mm)	26.96	27.73	25.91	20.45	23.21	29.82	29.99	28.97	27.53	19.17
b (mm)	7.04	23.8	11.13	8.32	11.14	14.02	11.08	8.04	10.13	9.37
c (mm)	4.08	3.57	5.16	4.03	4.23	5.13	3.15	4.02	6.0	3.12
Ψ	0.3	0.4	0.4	0.4	0.4	0.4	0.3	0.3	0.4	0.4

a, the longest particle diameter; b, the intermediate particle diameter; c, the shortest particle diameter; Ψ, sphericity.
